# Comparative Analysis of Chemical Composition and Antibacterial Activity of *Artemisia argyi* and *Artemisia lavandulifolia* Hydrosols

**DOI:** 10.1002/cbdv.202502063

**Published:** 2026-09-30

**Authors:** Xiaoqing Gao, Yuxin Li, Ting Fang, Wenjie Jiang

**Affiliations:** ^1^ School of Pharmaceutical Sciences Shandong University Jinan China; ^2^ School of Pharmacy Shandong University of Traditional Chinese Medicine (TCM) Jinan China

**Keywords:** antibacterial activity, *Artemisia argyi*, *Artemisia lavandulifolia*, chemical composition, GC–MS, hydrosol

## Abstract

Hydrosols from *Artemisia argyi* and *A. lavandulifolia* collected across different regions and seasons in Shandong Province, China, were comparatively analyzed for chemical composition and antibacterial activity. GC–MS results showed that *A. lavandulifolia* hydrosol contained higher levels of terpinen‐4‐ol and α‐terpineol and exhibited a chemical profile distinct from *A. argyi*. Variations in key volatile compounds suggested influences of harvest season and geographic origin. Antibacterial assays against five bacterial strains showed that both hydrosols inhibited *Escherichia coli* ATCC 25922 and *Proteus vulgaris* ATCC 49132, with *A. lavandulifolia* displaying stronger activity. Multivariate analyses, including HCA, PCA, and heatmaps, further supported species‐related differences with partial sample overlap. These findings highlight the compositional and bioactive diversity of *Artemisia* hydrosols and their potential as natural antimicrobial agents.

## Graphical abstract

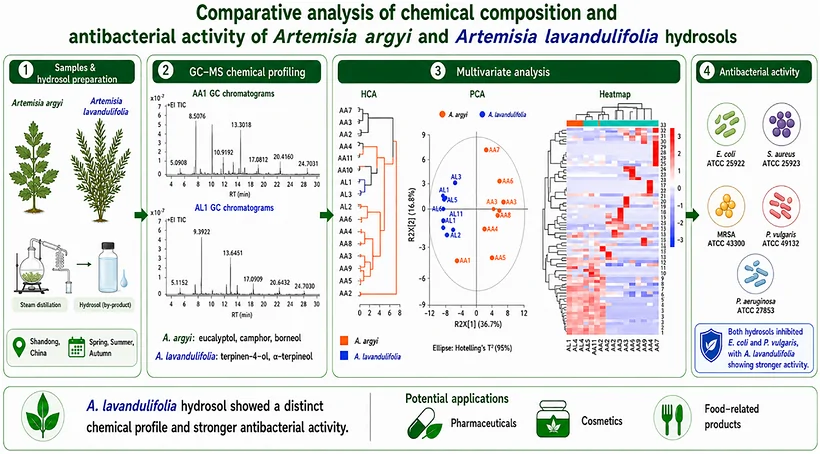

## 1 Introduction

Hydrosols, also known as floral waters or distillates, are by‐products obtained during the steam distillation of plant materials for essential oil extraction. These solutions contain water‐soluble volatile compounds and often retain several biological properties associated with their parent essential oils, including antioxidant, antimicrobial, and anti‐inflammatory effects [1, 2, 3, 4]. Recently, there has been renewed interest in hydrosols, spurred by their potential in cosmetic, food, and pharmaceutical applications [5, 6, 7].

*Artemisia argyi* and *A. lavandulifolia* are medicinal plants from the Asteraceae family, widely distributed across Asia [8]. *A. argyi*, commonly known as Chinese mugwort, is extensively used in traditional Chinese medicine for its reputed effects in warming meridians, dispelling cold, and alleviating inflammation and allergic responses [9, 10]. Its essential oil is rich in compounds such as eucalyptol, camphor, and borneol—well‐known for their antimicrobial and antioxidant activities [11, 12]. While the essential oil of *A. argyi* has been well‐studied, investigations into its hydrosol remain limited.

*A. lavandulifolia*, a closely related species, shares similar medicinal attributes and has been found to contain key volatiles like terpinen‐4‐ol and *α*‐terpineol in its essential oil [13, 14, 15]. However, detailed studies focusing on its hydrosol's chemical profile and antimicrobial potential are sparse, leaving an important research gap.

Moreover, environmental variables such as geographical origin and seasonal timing are known to influence the phytochemical profiles of plant‐based products [16, 17]. Previous research has demonstrated substantial chemical variability in *Artemisia* essential oils depending on location, climate, and harvest time [18, 19]. Despite this, few studies have systematically examined how such spatiotemporal factors affect hydrosol composition and associated bioactivities.

Recent investigations have advanced the understanding of *Artemisia* essential oils and related hydrolates. However, compared with essential oils, *Artemisia* hydrosols remain relatively underinvestigated despite their emerging commercial and therapeutic value [20, 21, 22]. Critically, although natural hydrolates have been investigated for their chemical composition and antimicrobial activity, studies on *Artemisia* hydrosols remain limited and still lack integrated evaluation of species‐specific, geographic, and seasonal influences, particularly in relation to antibacterial activity [23]. Moreover, although antimicrobial mechanisms of *Artemisia* essential oils have been increasingly elucidated, including membrane disruption and broad‐spectrum activity against Gram‐positive and Gram‐negative bacteria, such mechanistic insights are rarely extended to hydrosol systems or drug‐resistant pathogens.

To address these knowledge gaps, the present study offers a multi‐factorial comparative analysis of the chemical constituents and antibacterial activities of hydrosols derived from *A. argyi* and *A. lavandulifolia*. We hypothesized that species identity is an important factor associated with hydrosol chemotype, whereas geographic origin and harvest season may contribute to variation in volatile abundance and antibacterial activity. By evaluating samples harvested across different locations and seasons, this work explores the associations among environmental variability, chemical diversity, and antibacterial activity, providing foundational insight for the standardization and optimized use of *Artemisia* hydrosols in natural product development.

## 2 Results and Discussion

### 2.1 Chemical Composition of *Artemisia* Hydrosols

GC–MS analysis revealed clear differences in the chemical profiles of hydrosols derived from *A. argyi* and *A. lavandulifolia*. A total of 59 compounds were identified in *A. argyi* hydrosols, with eucalyptol, (+)‐camphor, and endo‐borneol as the major constituents. In contrast, *A. lavandulifolia* hydrosols were characterized by relatively high proportions of terpinen‐4‐ol and α‐terpineol (Supporting Information, Table 1). The total alcohol content was also higher in *A. lavandulifolia* than in *A. argyi*, suggesting a more oxygenated and water‐compatible volatile profile in this species.

These findings indicate that botanical origin is an important factor influencing the chemical composition of *Artemisia* hydrosols. Previous studies have mainly focused on essential oils, whereas hydrosols represent the aqueous distillate fraction and may selectively retain more water‐soluble volatile compounds [24, 25, 26, 27]. Several major compounds detected in *A. argyi* hydrosol, including eucalyptol, camphor, and borneol, are consistent with previously reported volatile profiles of this species [28, 29, 30]. For *A. lavandulifolia*, the predominance of terpinen‐4‐ol and α‐terpineol was broadly consistent with earlier reports on its volatile constituents. In addition, the species‐specific occurrence of α‐asarone in *A. lavandulifolia* hydrosol may serve as a potential chemical marker for distinguishing it from *A. argyi*, although further confirmation using authentic standards and larger sample sets is required.

**FIGURE 1:**
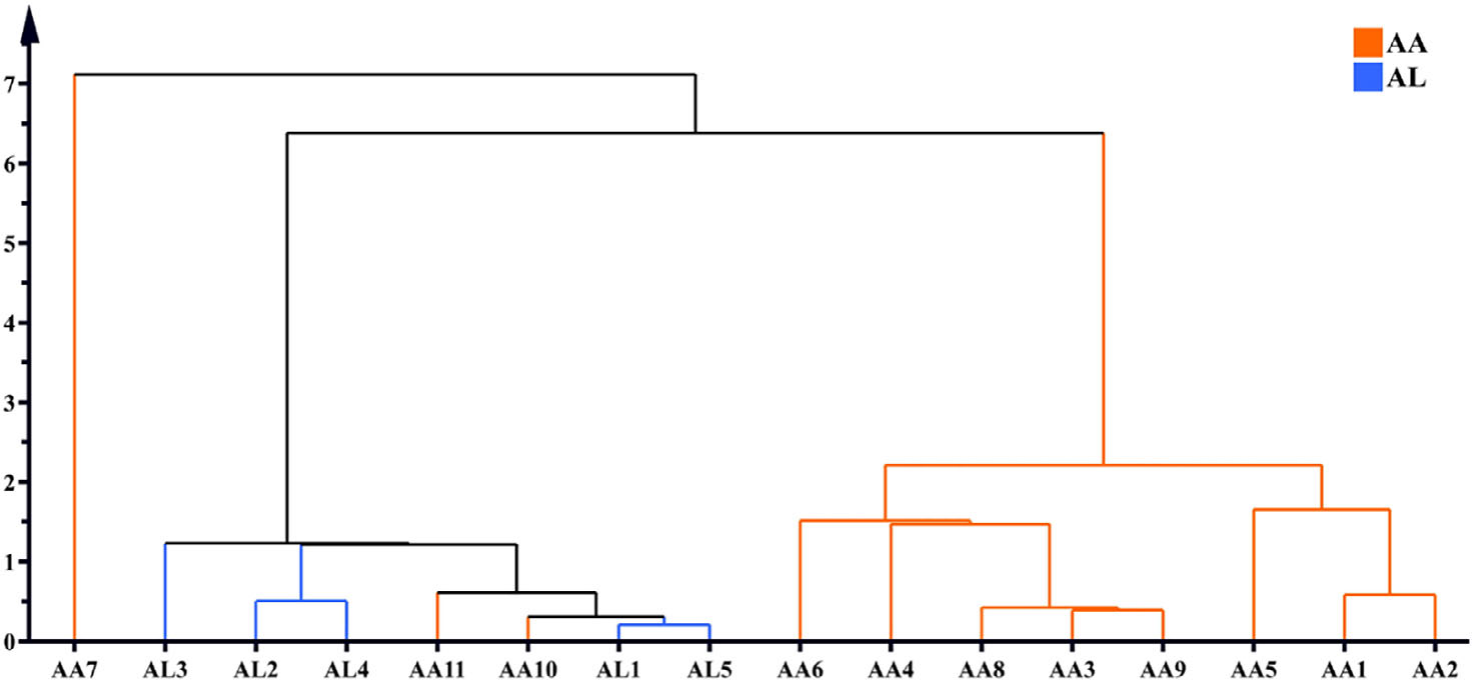
Hierarchical cluster analysis (HCA) of *Artemisia argyi* (AA) and *Artemisia lavandulifolia* (AL) hydrosol samples based on chemical composition, showing sample‐level similarities and differences between the two species.

**FIGURE 2:**
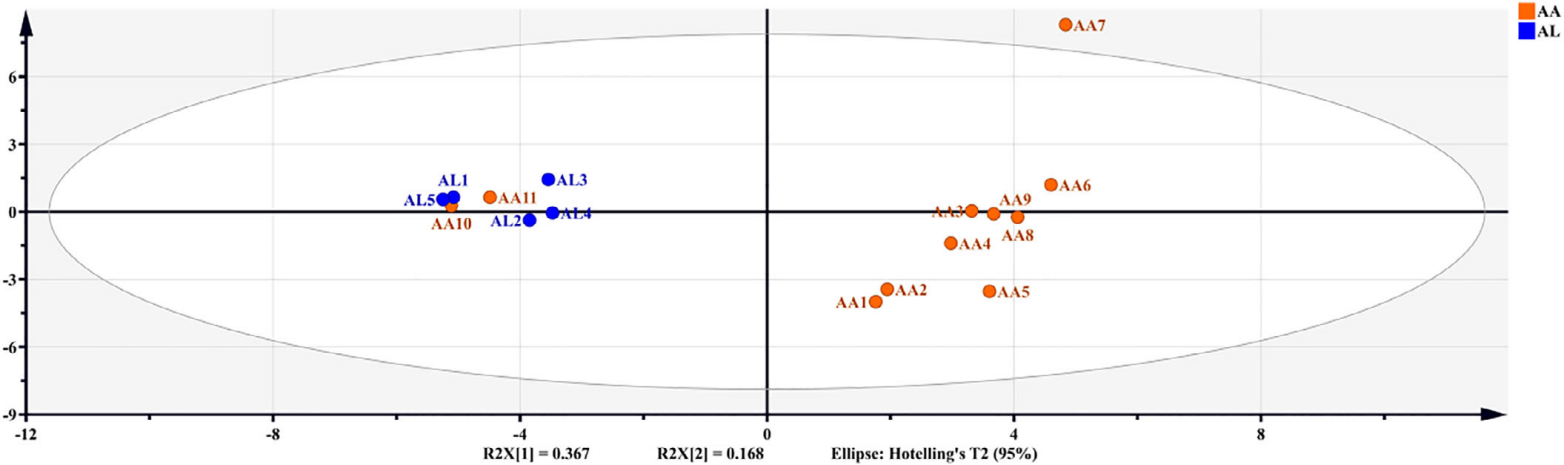
Principal component analysis (PCA) score plot depicting metabolic variation between *Artemisia argyi* (AA) and *Artemisia lavandulifolia* (AL) hydrosol samples. The plot shows species‐associated separation with partial overlap among individual samples.

**FIGURE 3:**
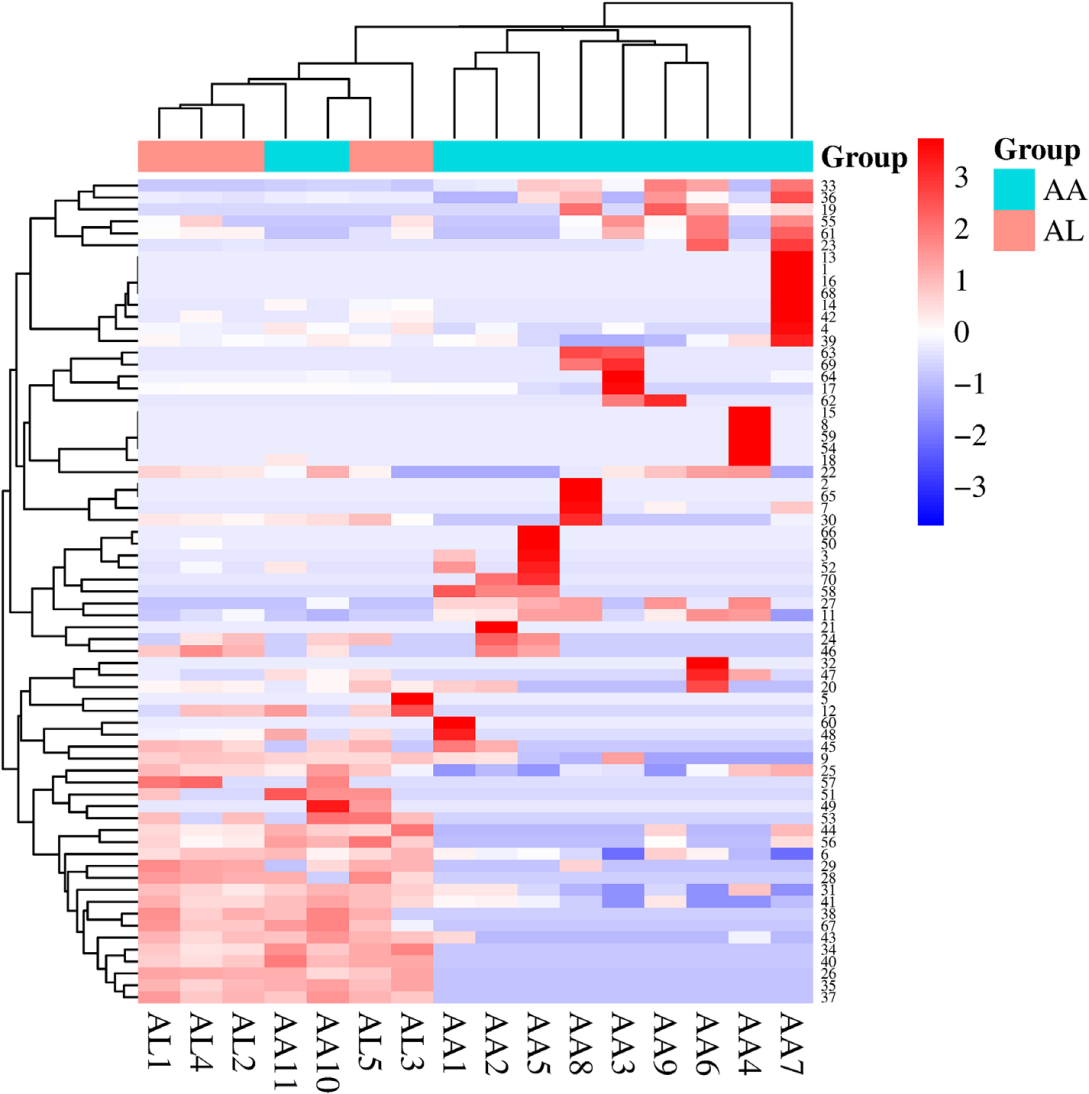
Heatmap representing the relative abundance of detected volatile compounds across *Artemisia* hydrosol samples. Rows correspond to metabolites (z‐score normalized), and columns represent samples grouped by cultivar: *Artemisia argyi* (AA, cyan) and *Artemisia lavandulifolia* (AL, red). Hierarchical clustering of both metabolites (left dendrogram) and samples (top dendrogram) illustrates patterns of co‐abundance and sample similarity. Color intensity scales from blue (low abundance) to red (high abundance).

Hydrosol composition also varied among samples collected from different geographic origins and harvest seasons. Differences in eucalyptol levels and alcohol content were observed among Zouping, Jinan, and Liaocheng samples, as well as between seasonal collections. These variations suggest that environmental factors and plant developmental stage may contribute to secondary metabolite accumulation [16]. However, because the sampling design was not fully balanced across species and locations, these geographic and seasonal effects should be interpreted cautiously.

### 2.2 Antibacterial Activity of Hydrosols

Both *A. argyi* and *A. lavandulifolia* hydrosols showed antibacterial activity against *Escherichia coli* ATCC 25922 and *Proteus vulgaris* ATCC 49132, with *A. lavandulifolia* generally displaying stronger inhibitory effects. Sample AL1 showed activity against all tested strains except MRSA ATCC 43300, whereas activity against *Pseudomonas aeruginosa* ATCC 27853 was limited to AA1, AA9, and AL1 at the undiluted concentration of 100% (v/v). MBC results further confirmed bactericidal activity mainly against *P. vulgaris* and *E. coli*. AL1 also displayed bactericidal effects against *Staphylococcus aureus* ATCC 25923 and Gram‐negative strains, while most samples showed no detectable MBC against MRSA ATCC 43300 or *P. aeruginosa* ATCC 27853 (Table 2).

**TABLE 1:** Composition of *Artemisia argyi* and A*rtemisia lavandulifolia* hydrosol.

No.	RT	RI	Compounds	Molecular formula	Molecular weight	M±SD (Relative content%)															
						AA1	AA2	AA3	AA4	AA5	AA6	AA7	AA8	AA9	AA10	AA11	AL1	AL2	AL3	AL4	AL5
1	4.43	750	Pantothenic acid	C_19_H_17_NO_5_	339	—	—	—	—	—	—	—	—	—	—	—	—	—	3.20 ± 0.54	—	—
2	4.77	845	2‐Hexenal, (E)‐	C_6_H_10_O	98	—	—	—	—	—	—	—	—	—	—	—	—	—	—	0.24 ± 0.02	—
3	5.11	1400	Cyclobutane,1,1‐dimethyl‐2‐octyl‐	C_14_H_28_	196	0.73 ± 0.01	—	—	—	—	—	—	—	—	—	—	2.46 ± 0.04	—	—	—	—
4	7.59	960	2‐Furancarboxaldehyde,5‐methyl‐	C_6_H_6_O_2_	110	—	—	—	—	—	—	—	—	0.41 ± 0.35	—	—	—	—	—	—	—
5	7.95	979	1‐Octen‐3‐ol	C_8_H_16_O	128	0.95 ± 0.04	0.81 ± 0.02	—	0.45 ± 0.09	0.95 ± 0.24	1.32 ± 0.01	1.08 ± 0.08	1.24 ± 0.08	1.33 ± 0.03	1.23 ± 0.18	1.11 ± 0.03	0.86 ± 0.02	0.96 ± 0.13	—	0.64 ± 0.05	1.18 ± 0.09
6	8.15	990	6‐Methyl‐5‐hepten‐2‐one	C_8_H_14_O	126	—	—	—	—	—	—	—	—	—	—	—	—	—	1.20 ± 0.17	4.04 ± 0.34	0.56 ± 0.07
7	8.24	1025	2,3‐Dehydro‐1,8‐cineole	C_10_H_16_O	152	—	—	—	0.49 ± 0.04	—	—	—	—	—	—	—	—	—	—	—	—
8	8.52	1040	Yomogi alcohol	C_10_H_18_O	154	21.01 ± 1.20	20.51 ± 0.26	32.19 ± 0.40	—	22.44 ± 0.17	23.54 ± 1.07	23.56 ± 1.71	24.77 ± 1.20	25.82 ± 0.56	25.98 ± 1.73	21.79 ± 0.08	5.30 ± 0.07	—	—	2.46 ± 0.39	—
9	8.94	1060	Isocineole	C_10_H_18_O	154	—	—	—	—	—	—	—	—	—	—	—	—	—	—	—	—
10	9.38	1030	Eucalyptol	C_10_H_18_O	154	21.64 ± 0.49	22.85 ± 0.21	13.10 ± 0.28	33.90 ± 0.33	7.99 ± 0.09	10.87 ± 0.43	10.89 ± 0.90	17.86 ± 1.02	11.68 ± 0.11	14.41 ± 0.91	11.67 ± 0.18	33.04 ± 0.24	35.99 ± 1.06	4.41 ± 0.21	33.42 ± 0.16	22.11 ± 0.26
11	9.56	1050	(S)‐2,5‐Dimethyl‐3‐vinylhex‐4‐en‐2‐ol	C_10_H_18_O	154	—	—	—	—	—	0.34 ± 0.10	—	0.24 ± 0.08	0.53 ± 0.05	0.25 ± 0.05	0.21 ± 0.01	—	—	—	—	—
12	9.67	1110	3‐Cyclohexen‐1‐one,3,5,5‐trimethyl‐	C_9_H_14_O	138	—	—	—	—	—	—	—	—	—	—	—	—	—	0.84 ± 0.17	—	—
13	9.78	1040	Phenylacetaldehyde	C_8_H_8_O	120	—	—	—	—	—	0.69 ± 0.04	—	—	0.57 ± 0.28	—	0.27 ± 0.01	—	—	6.28 ± 1.34	—	—
14	10.45	1065	*trans*‐Sabinene Hydrate	C_10_H_18_O	154	—	—	—	3.31 ± 0.03	—	—	—	—	—	—	—	—	—	—	—	—
15	10.61	1070	*cis*‐Linalool oxide (furanoïd)	C_10_H_18_O_2_	170	—	—	—	—	—	—	—	—	—	—	—	—	—	0.56 ± 0.04	—	—
16	10.92	1090	*Artemisia* alcohol	C_10_H_18_O	154	2.97 ± 0.10	3.06 ± 0.12	22.88 ± 0.19	—	3.48 ± 0.06	3.47 ± 0.02	3.21 ± 0.34	3.58 ± 0.26	3.75 ± 0.06	3.71 ± 0.05	3.00 ± 0.28	0.70 ± 0.05	—	—	—	—
17	11.35	1120	*cis*‐Sabinene	C_10_H_18_O	154	—	—	—	4.77 ± 0.04	—	0.76 ± 1.07	—	—	—	—	—	—	—	—	—	—
18	11.50	1102	Linalool	C_10_H_14_O	150	—	—	—	0.41 ± 0.08	—	—	—	—	—	—	—	—	1.09 ± 0.05	0.65 ± 0.15	1.60 ± 0.15	1.77 ± 0.04
19	11.56	1105	*α*‐Thujone	C_10_H_16_O	152	2.60 ± 0.05	2.88 ± 0.03	—	—	1.72 ± 0.06	0.98 ± 1.14	1.70 ± 0.09	1.85 ± 0.17	1.96 ± 0.21	1.94 ± 0.21	2.87 ± 0.25	—	5.71 ± 0.09	—	—	—
20	11.99	1070	Cyclohexene, 1,5,5‐trimethyl‐6‐methylene‐	C_10_H_16_	152	—	0.27 ± 0.02	—	—	—	—	—	—	—	—	—	—	—	—	—	—
21	12.06	1135	*cis*‐p‐Menth‐2‐en‐1‐ol	C_10_H_18_O	154	—	—	0.75 ± 0.03	1.25 ± 0.02	1.11 ± 0.13	0.52 ± 0.11	0.89 ± 0.11	0.72 ± 0.14	—	0.76 ± 0.13	0.67 ± 0.07	—	1.21 ± 0.03	—	0.44 ± 0.07	0.97 ± 0.02
22	12.11	1140	2‐Pinen‐7‐one	C_10_H_14_O	150	—	—	—	—	—	—	—	0.09 ± 0.08	—	—	—	—	3.68 ± 0.37	4.50 ± 0.14	—	0.18 ± 0.02
23	12.43	1160	*trans*‐Chrysanthenol	C_10_H_16_O	152	—	0.32 ± 0.01	—	—	0.15 ± 0.13	—	—	0.18 ± 0.01	—	0.12 ± 0.11	0.18 ± 0.02	0.25 ± 0.03	—	—	—	—
24	12.56	1155	2‐Cyclohexen‐1‐ol,1‐methyl‐4‐(1‐methylethyl)‐, *cis*‐	C_10_H_18_O	154	—	0.28 ± 0.01	0.64 ± 0.01	1.33 ± 0.22	1.66 ± 0.01	1.01 ± 0.00	1.43 ± 0.05	1.16 ± 0.08	0.74 ± 0.05	1.16 ± 0.08	1.29 ± 0.08	—	0.77 ± 0.04	1.50 ± 0.03	0.68 ± 0.06	—
25	12.67	1145	(+)‐Camphor	C_10_H_16_O	152	9.64 ± 0.21	9.83 ± 0.10	—	16.23 ± 0.31	13.68 ± 0.62	12.80 ± 0.26	14.16 ± 0.63	13.12 ± 0.98	13.89 ± 0.70	13.30 ± 1.04	10.61 ± 0.18	13.04 ± 0.29	3.29 ± 0.12	3.45 ± 0.06	14.22 ± 0.02	15.40 ± 0.20
26	12.80	1350	2‐Allyl‐2‐bornanol	C_12_H_22_O	194	—	—	—	—	—	0.69 ± 0.01	0.82 ± 0.13	0.69 ± 0.02	0.49 ± 0.03	0.76 ± 0.03	0.89 ± 0.04	—	—	—	—	—
27	13.06	1165	Bicyclo[3.1.0]hexan‐2‐one,5‐(1‐methylethylethyl)‐	C_10_H_16_O	152	—	—	—	—	0.18 ± 0.16	—	0.33 ± 0.08	0.27 ± 0.05	0.25 ± 0.01	0.28 ± 0.03	0.26 ± 0.01	—	—	—	0.19 ± 0.01	—
28	13.22	1175	*cis*‐Chrysanthenol	C_10_H_16_O	152	—	—	—	—	0.79 ± 0.06	0.69 ± 0.00	0.73 ± 0.07	0.57 ± 0.01	0.50 ± 0.03	0.64 ± 0.08	1.12 ± 0.01	—	—	0.37 ± 0.04	2.49 ± 0.03	—
29	13.31	1167	endo‐Borneol	C_10_H_18_O	154	12.38 ± 0.26	12.47 ± 0.06	—	16.18 ± 0.07	17.49 ± 0.12	14.97 ± 0.13	16.37 ± 0.65	12.77 ± 0.82	15.11 ± 0.43	14.60 ± 0.64	16.45 ± 0.18	6.75 ± 0.05	—	—	3.53 ± 0.05	6.72 ± 0.07
30	13.35	1190	(‐)‐*α*‐Terpineol	C_10_H_18_O	154	—	—	—	—	—	—	—	—	—	—	—	—	1.49 ± 0.03	—	—	—
31	13.64	1176	Terpinen‐4‐ol	C_10_H_18_O	154	7.78 ± 0.15	8.21 ± 0.07	8.93 ± 0.03	3.93 ± 0.12	5.75 ± 0.08	5.32 ± 0.02	5.07 ± 0.32	5.02 ± 0.19	5.04 ± 0.06	4.95 ± 0.33	5.59 ± 0.12	14.31 ± 0.28	17.42 ± 0.27	21.88 ± 0.97	13.81 ± 0.19	20.90 ± 0.25
32	13.88	1195	*p*‐Cymen‐8‐ol	C_10_H_14_O	150	—	—	—	—	0.34 ± 0.09	0.51 ± 0.08	0.34 ± 0.08	0.27 ± 0.03	0.54 ± 0.05	0.26 ± 0.08	0.43 ± 0.06	—	—	—	—	—
33	13.93	1200	*trans*‐p‐Mentha‐1(7),8‐dien‐2‐ol	C_10_H_16_O	152	—	—	—	—	0.38 ± 0.03	0.34 ± 0.04	0.33 ± 0.07	0.32 ± 0.03	0.37 ± 0.03	0.25 ± 0.03	0.31 ± 0.05	—	—	—	—	—
34	14.03	1192	*α*‐Terpineol	C_10_H_18_O	154	—	—	—	2.94 ± 0.09	4.75 ± 0.21	4.42 ± 0.20	4.38 ± 0.04	3.76 ± 0.22	4.30 ± 0.07	4.20 ± 0.21	4.53 ± 0.15	8.53 ± 0.11	6.51 ± 0.59	19.55 ± 0.80	11.08 ± 0.05	13.94 ± 0.27
35	14.18	1190	Myrtenol	C_10_H_16_O	152	—	—	—	—	0.70 ± 0.05	0.48 ± 0.17	0.68 ± 0.11	0.56 ± 0.05	0.49 ± 0.02	0.49 ± 0.09	0.57 ± 0.06	—	—	—	—	—
36	14.30	1230	(‐)‐*cis*‐Isopiperitenol	C_10_H_16_O	152	—	—	—	—	0.44 ± 0.13	0.30 ± 0.42	0.41 ± 0.07	0.34 ± 0.09	—	0.25 ± 0.08	0.33 ± 0.07	—	—	—	—	—
37	14.54	1205	Verbenone	C_10_H_14_O	150	0.85 ± 0.04	0.95 ± 0.02	—	1.21 ± 0.03	1.01 ± 0.08	0.77 ± 0.05	0.93 ± 0.09	0.78 ± 0.13	0.64 ± 0.03	0.71 ± 0.07	0.91 ± 0.07	0.44 ± 0.04	0.75 ± 0.07	3.22 ± 0.20	—	0.10 ± 0.03
38	14.66	1250	3, *Trans*‐(1,1‐dimethylethyl)‐4, *cis*‐methoxycyclohexan‐1‐ol	C_11_H_22_O_2_	186	—	—	—	—	0.52 ± 0.04	0.77 ± 0.12	0.42 ± 0.07	0.45 ± 0.07	0.58 ± 0.06	0.36 ± 0.01	0.57 ± 0.03	—	—	—	—	—
39	14.82	1222	*trans*‐Carveol	C_10_H_16_O	152	3.16 ± 0.27	3.31 ± 0.06	—	—	5.43 ± 0.03	4.72 ± 0.36	5.16 ± 0.08	4.10 ± 0.43	3.99 ± 0.30	4.05 ± 0.21	4.81 ± 0.13	2.59 ± 0.33	—	1.19 ± 0.13	1.70 ± 0.02	3.67 ± 0.07
40	14.94	1300	(+)‐*cis*‐Verbenol, acetate	C_12_H_18_O_2_	194	—	—	—	—	—	—	—	—	0.13 ± 0.11	0.11 ± 0.18	0.11 ± 0.18	—	—	1.06 ± 0.24	—	—
41	15.07	1225	*cis*‐p‐Mentha‐1(7),8‐dien‐2‐ol	C_10_H_16_O	152	0.26 ± 0.02	—	—	0.14 ± 0.01	0.42 ± 0.01	0.31 ± 0.07	0.35 ± 0.03	0.33 ± 0.04	0.31 ± 0.03	0.26 ± 0.05	0.35 ± 0.03	—	—	—	—	—
42	15.16	1223	*cis*‐Carveol	C_10_H_16_O	152	—	—	—	—	0.51 ± 0.03	0.64 ± 0.16	0.47 ± 0.13	0.41 ± 0.05	0.89 ± 0.06	0.38 ± 0.09	0.47 ± 0.04	—	—	0.60 ± 0.04	—	0.49 ± 0.06
43	15.50	1242	D‐Carvone	C_10_H_14_O	150	0.31 ± 0.09	0.23 ± 0.01	—	—	0.18 ± 0.16	—	0.21 ± 0.04	0.16 ± 0.14	—	0.20 ± 0.05	0.22 ± 0.01	—	—	—	—	—
44	15.80	1238	Piperitone	C_10_H_16_O	152	—	0.25 ± 0.01	—	—	0.11 ± 0.09	—	0.15 ± 0.00	0.18 ± 0.11	—	0.24 ± 0.09	—	0.20 ± 0.02	—	—	—	—
45	16.29	1245	(S)‐Isopiperitenone	C_10_H_14_O	150	—	—	—	0.68 ± 0.06	0.25 ± 0.23	0.40 ± 0.09	0.11 ± 0.19	—	—	—	0.41 ± 0.15	—	1.45 ± 0.29	—	—	—
46	16.63	1351	p‐Menth‐1‐en‐3,8‐diol	C_10_H_18_O_2_	170	0.82 ± 0.06	—	—	—	—	0.38 ± 0.01	0.06 ± 0.10	0.14 ± 0.04	—	0.09 ± 0.08	0.23 ± 0.02	—	—	—	—	—
47	16.77	1450	Limonen‐6‐ol, pivalate	C_15_H_24_O_2_	236	—	—	—	—	0.24 ± 0.21	—	—	—	—	—	0.12 ± 0.21	—	—	—	—	—
48	16.96	1290	p‐Mentha‐1,8‐dien‐7‐ol	C_10_H_16_O	152	—	—	—	—	0.26 ± 0.23	0.36 ± 0.07	0.17 ± 0.14	—	—	—	0.26 ± 0.25	—	—	—	—	—
49	17.09	1300	p‐menthane‐1,8‐diol	C_10_H_20_O_2_	172	1.38 ± 0.10	—	—	—	—	0.54 ± 0.12	—	—	—	0.23 ± 0.09	—	2.64 ± 0.10	—	—	—	—
50	17.11	1550	4‐(2,2,6‐Trimethyl‐bicyclo[4.1.0]hept‐1‐yl)‐butan‐2‐one	C_14_H_24_O	208	—	—	—	—	0.50 ± 0.14	—	0.29 ± 0.07	0.30 ± 0.02	0.30 ± 0.05	—	0.49 ± 0.13	—	—	—	—	—
51	17.42	950	1,3‐Cyclopentadiene,5,5‐dimethyl‐1‐ethyl‐	C_9_H_14_	122	—	—	—	1.58 ± 0.17	—	—	—	—	—	—	—	—	—	—	—	—
52	18.26	1360	Eugenol	C_10_H_12_O_2_	164	—	—	—	—	3.88 ± 0.33	4.50 ± 0.14	3.05 ± 0.18	2.40 ± 0.25	3.25 ± 0.08	2.11 ± 0.24	5.61 ± 0.19	—	—	2.74 ± 0.11	—	1.90 ± 0.44
53	18.42	1445	Acetyl isoeugenol	C_10_H_12_O_2_	164	—	—	—	—	0.10 ± 0.17	—	0.11 ± 0.20	—	—	0.12 ± 0.20	—	—	—	—	—	—
54	18.65	1310	Sobrerol	C_10_H_18_O_2_	170	0.31 ± 0.04	0.24 ± 0.02	—	—	—	—	—	—	—	—	—	0.24 ± 0.02	—	—	—	—
55	19.05	1405	*cis*‐jasmone	C_11_H_16_O	164	—	—	—	0.17 ± 0.01	—	—	—	—	—	—	—	—	—	—	—	—
56	21.84	1575	Spathulenol	C_15_H_24_O	220	—	—	0.16 ± 0.02	—	—	—	—	—	—	—	—	—	—	—	—	0.24 ± 0.00
57	22.32	1625	*α*‐Asarone	C_12_H_16_O_3_	208	—	—	2.48 ± 0.11	—	—	—	—	—	—	—	—	—	—	—	2.64 ± 0.04	—
58	22.85	1640	Neointermedeol	C_15_H_26_O	222	—	—	9.03 ± 0.02	—	0.55 ± 0.11	0.33 ± 0.04	0.32 ± 0.03	0.33 ± 0.01	—	0.30 ± 0.07	0.36 ± 0.03	—	—	0.50 ± 0.00	—	—
59	22.95	1655	Patchouli alcohol	C_15_H_26_O	222	—	—	—	—	—	—	—	—	—	—	—	—	—	—	0.17 ± 0.01	—
60	23.63	1590	Isoaromadendrene epoxide	C_15_H_24_O	220	—	—	—	—	—	—	—	—	—	—	—	0.10 ± 0.00	—	—	—	—
61	23.23	1750	Kessanyl acetate	C_17_H_28_O_3_	280	—	—	—	—	0.44 ± 0.05	0.39 ± 0.01	0.40 ± 0.08	0.27 ± 0.03	0.10 ± 0.08	0.27 ± 0.00	0.29 ± 0.03	—	—	—	—	—
62	23.29	1850	10‐Methyl–8‐tetradecen‐1‐ol acetate	C_17_H_32_O_2_	268	—	—	—	—	—	—	—	—	—	—	—	—	—	0.56 ± 0.07	—	—
63	23.94	2000	Isocalamenediol	C_15_H_26_O_2_	238	—	—	0.32 ± 0.04	—	—	—	—	—	—	—	—	—	—	—	0.22 ± 0.01	—
64	24.70	2100	Cryptomeridiol	C_15_H_28_O_2_	240	—	0.15 ± 0.00	—	—	—	—	—	—	—	—	—	0.21 ± 0.01	—	—	—	—
Terpenes						43.61	44.57	34.65	63.44	38.39	37.42	40.60	45.66	38.31	42.60	39.61	71.26	77.16	53.08	78.06	75.51
Alcohols						41.60	40.72	54.68	22.08	53.99	53.65	52.49	49.45	53.15	51.99	51.98	16.69	1.51	1.24	10.80	11.77
Esters						—	—	—	—	0.44	0.39	0.40	0.27	0.23	0.38	0.40	—	—	1.62	—	—
Aldehydes						—	0.38	0.44	—	0.41	1.40	0.39	0.23	1.75	0.29	0.51	—	—	9.64	0.24	—
Ethers						—	—	0.71	—	—	—	0.28	0.17	0.38	0.42	0.08	—	0.90	0.92	0.24	0.29
Ketones						0.85	0.95	—	1.38	1.19	0.77	1.26	1.05	0.89	0.99	1.17	0.44	0.75	5.26	4.23	0.66
Phenols						—	—	—	—	3.98	4.50	3.16	2.40	3.25	2.23	5.61	—	—	2.74	—	1.90
Others						0.73	—	—	2.07	—	—	—	—	—	0.07	—	3.27	—	3.76	—	—
Total						86.79	86.62	90.48	88.97	98.4	98.13	98.58	99.23	97.96	98.97	99.36	91.66	80.32	78.26	93.57	90.13

*Note*: Data are reported as the mean ± standard deviation (*n* = 3). The relative content percentage of each compound was calculated using the area normalization method.

Abbreviations: AA: *Artemisia argyi*; AL: *Artemisia lavandulifolia*.

**TABLE 2:** MIC and MBC values of *Artemisia argyi* and *Artemisia lavandulifolia* hydrosol for five bacterial strains.

Sample Strains	AA1		AA2		AA3		AA4		AA5		AA6		AA7		AA8		AA9		AA10		AA11		AL1		AL2		AL3		AL4		AL5	
	MIC	MBC	MIC	MBC	MIC	MBC	MIC	MBC	MIC	MBC	MIC	MBC	MIC	MBC	MIC	MBC	MIC	MBC	MIC	MBC	MIC	MBC	MIC	MBC	MIC	MBC	MIC	MBC	MIC	MBC	MIC	MBC
EC	100	100	100	—	100	—	100	—	100	>100	100	>100	100	>100	100	>100	100	>100	100	>100	100	>100	100	100	100	—	100	—	>100	—	>100	—
MRSA	>100	—	>100	—	>100	—	>100	—	>100	—	>100	—	>100	—	>100	—	>100	—	>100	—	>100	—	>100	—	>100	—	>100	—	>100	—	>100	—
PA	100	—	>100	—	>100	—	>100	—	>100	—	>100	—	>100	—	>100	—	100	>100	>100	—	>100	—	100	—	>100	—	>100	—	>100	—	>100	—
PV	50	100	>100	—	100	—	>100	—	>100	—	>100	—	>100	—	>100	—	100	100	>100	—	>100	—	50	100	>100	—	100	100	100	100	>100	—
SA	>100	—	>100	—	>100	—	>100	—	100	>100	100	>100	100	>100	>100	—	100	>100	>100	—	100	>100	50	100	>100	—	100	—	>100	—	>100	—

*Note*: MIC and MBC values are expressed as hydrosol concentration, % (v/v). “>100” indicates no inhibitory or bactericidal effect at the highest tested concentration, namely undiluted hydrosol at 100% (v/v). “–” indicates that bactericidal activity was not detected at the tested concentrations or was not determined because no inhibitory endpoint was observed.

Abbreviations: AA: *Artemisia argyi*; AL: *Artemisia lavandulifolia*; MIC: minimum inhibitory concentration; MBC: minimum bactericidal concentration; EC: *Escherichia coli* ATCC 25922; MRSA: methicillin‐resistant *Staphylococcus aureus* ATCC 43300; PA: *Pseudomonas aeruginosa* ATCC 27853; PV: *Proteus vulgaris* ATCC 49132; SA: *Staphylococcus aureus* ATCC 25923.

The stronger antibacterial activity of *A. lavandulifolia* hydrosol may be associated with its higher content of oxygenated monoterpenes, especially terpinen‐4‐ol and α‐terpineol. Johansen et al. [31] reported that terpinen‐4‐ol and α‐terpineol can act synergistically against ESKAPE pathogens by reducing MIC and MBC values and enhancing membrane disruption. Although the present study did not directly test purified compounds or defined combinations, the results suggest that naturally occurring mixtures of oxygenated volatile compounds in *A. lavandulifolia* hydrosol may contribute to its antibacterial potential. The relatively limited activity against MRSA and *P. aeruginosa* indicates that the antibacterial spectrum of these hydrosols remains restricted and may require further formulation optimization or compound enrichment. The observed activity against Gram‐negative strains is also consistent with previous reports on antibacterial effects of *Artemisia*‐derived products [32].

### 2.3 Multivariate Analysis of Chemical Profiles

HCA, PCA, and heatmap analyses further supported the chemical differences between *A. argyi* and *A. lavandulifolia* hydrosols. HCA showed partial species‐associated clustering, although several samples from the two species were intermingled. PCA showed that PC1 and PC2 explained 36.8% and 16.8% of the total variation, respectively. Most AA and AL samples were separated, while AA10 and AA11 were closer to the AL cluster and AA7 appeared as an outlier, indicating sample‐specific chemical variation. The heatmap also revealed distinct abundance patterns of major volatile compounds between AA and AL samples (Figures 1, 2, 3).

These multivariate results suggest that species identity is an important determinant of hydrosol chemotype, but it is not the only factor shaping chemical composition. Geographic origin, harvest season, and sample‐specific variation may also influence volatile profiles [33]. Compounds such as α‐asarone, terpinen‐4‐ol, and α‐terpineol may therefore be useful as candidate markers for species differentiation and quality control, but their diagnostic value should be validated in broader sample sets.

### 2.4 Application Implications

The chemical diversity and antibacterial activity observed in this study indicate that *Artemisia* hydrosols have potential applications in food preservation, personal care, pharmaceutical, and cosmetic products [34, 35, 36]. Compared with essential oils, hydrosols are aqueous, milder, and easier to incorporate into water‐based formulations. However, the compositional variability associated with species, harvest season, and geographic source highlights the need for standardization of plant materials, collection time, and distillation procedures. Further studies should focus on compound‐level validation, mechanism‐oriented antibacterial assays, and formulation strategies to improve reproducibility and bioactivity.

## 3 Materials and Methods

### 3.1 Plant Materials

Fresh aerial parts (leaves and inflorescences) of *A. argyi* and *A. lavandulifolia* were collected from three distinct locations in Shandong Province, China: Zouping, Jinan, and Liaocheng. The samples were gathered during two seasonal windows: early summer (June, 2024) and late autumn (November, 2024), to capture temporal variation in phytochemical profiles (Table 3). Plant identity was verified by Professor Lingchuan Xu from Shandong University of Traditional Chinese Medicine (TCM), China. Voucher specimens were deposited in the herbarium of Shandong University of TCM under the following accession numbers: STCM2024601–STCM2024616.

**TABLE 3:** Species and geographic locations of *Artemisia* hydrosol samples.

No.	Species	Abbr.	Origins	Latitude and longitude	Elevation (m)	Date of collection	Voucher number
1	*A. argyi*	AA1	Zouping, shandong	117°46′20.58″E 36°45′46.86″N	399	2024.06	STCM2024601
2	*A. argyi*	AA2	Zouping, shandong	117°46′20.58″E 36°45′46.86″N	399	2024.06	STCM2024602
3	*A. argyi*	AA3	Jinan, shandong	116°48′06″E 36°33′38.50″N	60	2024.10	STCM2024603
4	*A. argyi*	AA4	Liaocheng, shandong	116°15′10.53″E 36°52′54.49″N	30	2024.10	STCM2024604
5	*A. argyi*	AA5	Zouping, shandong	117°46′20.58″E 36°45′46.86″N	399	2024.11	STCM2024605
6	*A. argyi*	AA6	Zouping, shandong	117°46′20.58″E 36°45′46.86″N	399	2024.11	STCM2024606
7	*A. argyi*	AA7	Zouping, shandong	117°46′20.58″E 36°45′46.86″N	399	2024.11	STCM2024607
8	*A. argyi*	AA8	Zouping, shandong	117°46′20.58″E 36°45′46.86″N	399	2024.11	STCM2024608
9	*A. argyi*	AA9	Zouping, shandong	117°46′20.58″E 36°45′46.86″N	399	2024.11	STCM2024609
10	*A. argyi*	AA10	Zouping, shandong	117°46′20.58″E 36°45′46.86″N	399	2024.11	STCM2024610
11	*A. argyi*	AA11	Zouping, shandong	117°46′20.58″E 36°45′46.86″N	399	2024.11	STCM2024611
12	*A. lavandulifolia*	AL1	Zouping, shandong	117°46′20.58″E 36°45′46.86″N	399	2024.06	STCM2024612
13	*A. lavandulifolia*	AL2	Zouping, shandong	117°46′20.58″E 36°45′46.86″N	399	2024.11	STCM2024613
14	*A. lavandulifolia*	AL3	Zouping, shandong	117°46′20.58″E 36°45′46.86″N	399	2024.11	STCM2024614
15	*A. lavandulifolia*	AL4	Zouping, shandong	117°46′20.58″E 36°45′46.86″N	399	2024.11	STCM2024615
16	*A. lavandulifolia*	AL5	Zouping, shandong	117°46′20.58″E 36°45′46.86″N	399	2024.11	STCM2024616

Following collection, plant materials were air‐dried under shaded, ventilated conditions at 25°C ± 2°C for 7 days. The dried materials were then pulverized using a mechanical grinder to ensure homogeneity for subsequent distillation procedures.

### 3.2 Hydrosol Extraction

Hydrosols were obtained through steam distillation using a modified Clevenger‐type apparatus [37, 38]. For each distillation, 20 g of powdered plant material was combined with 200 mL of distilled water in a 1000 mL round‐bottom flask. To prevent bumping and enhance heating uniformity, zeolites were added to the suspension, which was then allowed to stand at room temperature for 2 h to facilitate proper hydration.

The mixture was heated and distilled for 2 h, beginning from the emergence of the first distillate drop. The distillation temperature was carefully maintained at 100°C. The resulting hydrosol, comprising the complete aqueous phase, was collected in amber glass bottles and stored at 4°C to minimize photodegradation and microbial contamination [38, 39]. No liquid‐liquid extraction or essential oil separation was performed, and no drying agents were employed, in order to preserve the native composition of the hydrosol. The hydrosol was used as the 100% concentration for subsequent experiments, and dilutions were prepared using sterile distilled water.

### 3.3 GC–MS Analysis

#### 3.3.1 GC Conditions

The chemical constituents of the hydrosols were characterized using an Agilent GC–MS system equipped with a DB‐5MS capillary column (30 m × 0.25 mm × 0.25 µm; stationary phase: 5% diphenyl / 95% dimethyl polysiloxane). Helium (99.999%) was used as the carrier gas at a constant flow rate of 1 mL min^−1^. Injection was conducted in split mode (10:1), with an injector temperature of 250°C and injection volume of 1 µL. The column temperature was programmed as follows: initial hold at 50°C for 2 min, followed by an increase to 120°C at 5°C min^−1^, then to 250°C at 10°C min^−1^ with a final hold for 2 min [40, 41].

#### 3.3.2 MS Conditions

Mass spectrometry was performed using electron impact (EI) ionization at 70 eV. The ion source temperature was maintained at 280°C, and spectra were acquired in the 50–500 m/z range. Retention indices (RI) were calculated using a homologous series of n‐alkanes (C_7_–C_30_, Sigma‐Aldrich, St. Louis, MO, USA) under the same chromatographic conditions. Compounds in the hydrosols were identified through spectral comparison with entries in the NIST mass spectral library and confirmed by calculating retention indices against known standards [42].

### 3.4 Antibacterial Assay

#### 3.4.1 Bacterial Strains and Culture Conditions

Five bacterial strains were selected to evaluate the antimicrobial properties of the hydrosols: *Pseudomonas aeruginosa* ATCC 27853, *Escherichia coli* ATCC 25922, Methicillin‐resistant *Staphylococcus, aureus* (MRSA) ATCC 43300, *Proteus vulgaris* ATCC 49132 and *Staphylococcus aureus* ATCC 25923. These strains were procured from the American Type Culture Collection (ATCC, Manassas, VA, USA). Prior to antimicrobial assays, all strains were cultured in Tryptic Soy Broth (TSB; Hopebio, Qingdao, China) at 35°C for 24 h and maintained on Tryptic Soy Agar (TSA) plates at 4°C for short‐term preservation [43].

#### 3.4.2 Inoculum Preparation

Bacterial suspensions were prepared by picking a single colony from an overnight TSA plate and suspending it in sterile 0.9% (w/v) NaCl solution. The turbidity was adjusted to 0.5 McFarland standard (equivalent to approximately 1.0 × 10^8^ colony‐forming units [CFU]/mL) using a densitometer (DensiCHEK Plus, bioMérieux, Marcy‐l'Étoile, France). The suspension was then diluted 1:100 in Mueller‐Hinton Broth (MHB; Hopebio, Qingdao, China) to achieve a final inoculum concentration of 1.0 × 10^6^ CFU/mL.

#### 3.4.3 Minimum Inhibitory Concentration (MIC) Determination

The MIC values were determined using the broth microdilution method according to the Clinical and Laboratory Standards Institute (CLSI) guidelines M07‐A10. Native hydrosol samples were used directly as 100% (v/v) stock solutions without concentration, extraction, or any solvent addition. The hydrosols were filter‐sterilized through 0.22 µm polyethersulfone (PES) membranes and serially diluted two‐fold in MHB in 96‐well microtiter plates (Corning, NY, USA) to achieve final concentrations ranging from 100% to 3.125% (v/v) (100%, 50%, 25%, 12.5%, 6.25%, 3.125%). PBS was added to the peripheral wells to minimize evaporation of the liquid. Subsequently, 100 µL of MHB medium was added to each experimental well. Each experiment includes the following controls: The antibiotic positive control consisted of 100 µL of streptomycin sulfate solution (Shanghai Sangong Biology, Beijing, China) and 20 µL of bacterial suspension. The negative control consisted of 100 µL of MHB, 100 µL of sterile distilled water, and 20 µL of bacterial suspension, and was used to confirm bacterial viability and normal growth in the absence of hydrosol or antibiotic treatment. The blank control consisted of 100 µL of MHB and 100 µL of sterile distilled water without bacterial suspension, and was used to confirm the sterility of the medium. The plates were incubated at 35°C for 24 h under aerobic conditions. Following incubation, 20 µL of 0.5% (w/v) 2,3,5‐triphenyltetrazolium chloride (TTC; Aladdin, Shanghai, China) solution, freshly prepared and filter‐sterilized, was added to each well. The plates were further incubated at 35°C for 2 h in the dark. Bacterial dehydrogenases reduce colorless TTC to red formazan in metabolically active cells; therefore, the MIC was defined as the lowest concentration of hydrosol that prevented the color change from colorless to red, as determined by visual inspection. All assays were performed in triplicate [44, 45].

#### 3.4.4 Minimum Bactericidal Concentration (MBC) Determination

The MBC was determined by subculturing 10 µL aliquots from all wells showing no visible growth and no color change (TTC‐negative) in the MIC assay onto fresh TSA plates. The plates were incubated at 35°C for 24 h. The MBC was defined as the lowest concentration of hydrosol that killed ≥ 99.9% of the initial inoculum (corresponding to ≤ 1 colony on the agar plate, or a reduction of ≥ 3 log10 CFU/mL).

### 3.5 Data Analysis

Each experiment was repeated three times, and the MIC/MBC values are expressed as hydrosol concentration (%, v/v). Using the Origin 2021 software, Ward's connection method and Euclidean distance were employed for hierarchical clustering analysis (HCA) of the samples to group them based on chemical composition. Principal component analysis (PCA) was conducted using the correlation matrix, and data were standardized using automatic scaling. A heatmap was generated using the same distance and connection parameters.

## 4 Conclusion

This study supports the hypothesis that hydrosols from *Artemisia argyi* and *Artemisia lavandulifolia* differ in chemical composition and antibacterial potential, and suggests that these differences may be influenced by plant species, geographic origin, and harvest season. *Artemisia lavandulifolia* hydrosol showed a distinct chemical profile characterized by relatively high levels of oxygenated monoterpenes, especially terpinen‐4‐ol and *α*‐terpineol, whereas *Artemisia argyi* hydrosol was mainly characterized by eucalyptol, camphor, and borneol.

The relatively stronger antibacterial activity of *Artemisia lavandulifolia* hydrosol may be associated with its oxygenated monoterpene‐rich composition, although the underlying antibacterial mechanisms require further validation. The species‐specific occurrence of *α*‐asarone in *Artemisia lavandulifolia* hydrosol may provide a potential chemical marker for quality control and botanical authentication, but this should be confirmed using authentic standards and larger sample sets. Overall, this study provides an integrated evaluation of plant species, geographic origin, harvest season, chemical composition, and antibacterial activity in *Artemisia* hydrosols, offering useful information for the standardized development of *Artemisia lavandulifolia* hydrosol as a natural antimicrobial material.

## Author Contributions

**Xiaoqing Gao**: experimental operation, data analysis, and writing – original manuscript. **Yuxin Li**: experimental operation, data analysis. **Ting Fang**: experimental operation, data analysis. **Wenjie Jiang**: review and editing, supervision, and conceptualization.

## Funding

This research was funded by the Cooperation Project for the Construction of the Full Industry Chain of Traditional Chinese Medicine (TCM) in Shandong Zouping Baiyun Mountain (2024) (761130).

## Conflicts of Interest

The authors declare no conflicts of interest.

## Supporting information




**Supporting File 1**: cbdv71591‐sup‐0001‐SuppMat.docx

## Data Availability

The data that support the findings of this study are available from the corresponding author upon reasonable request.
