# Supplementary material for: Comparative Analysis of Chemical Composition and Antibacterial Activity of Artemisia argyi and Artemisia lavandulifolia Hydrosols
Source: Chem Biodivers. 2026 Sep 30;23(10):e02063. doi: 10.1002/cbdv.202502063 (PMC13626857; doi:10.1002/cbdv.202502063)
Supplement: Supplementary file 1 [file CBDV-23-e02063-s001.docx]

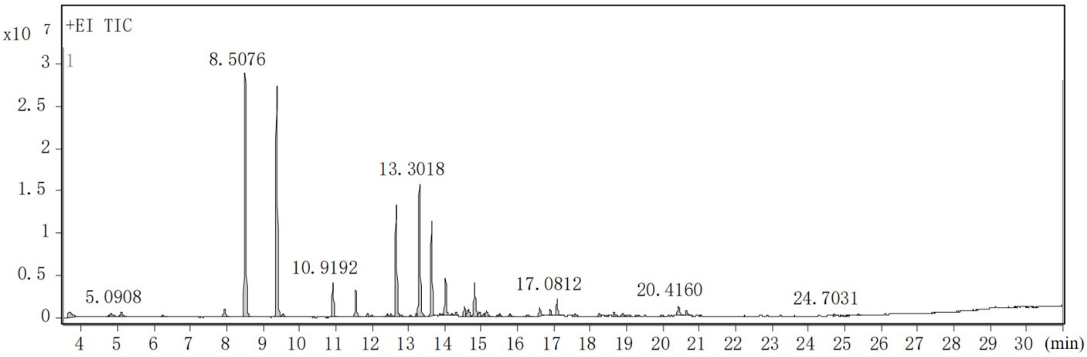

AA1 GC chromatogram

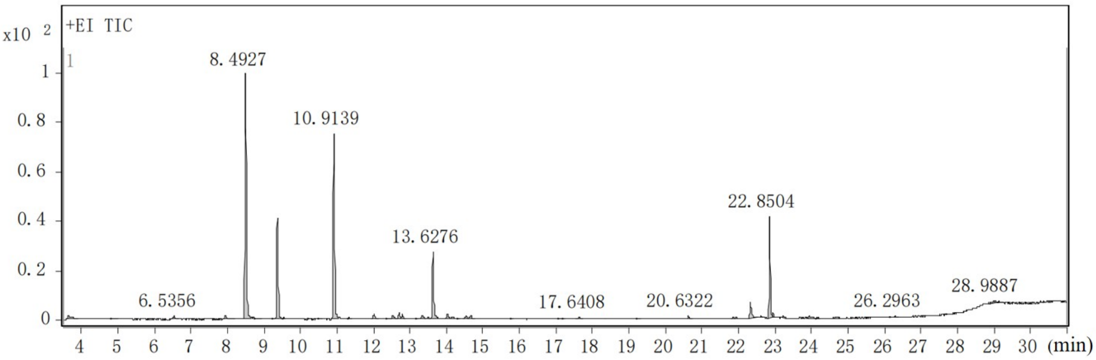

AA3 GC chromatogram

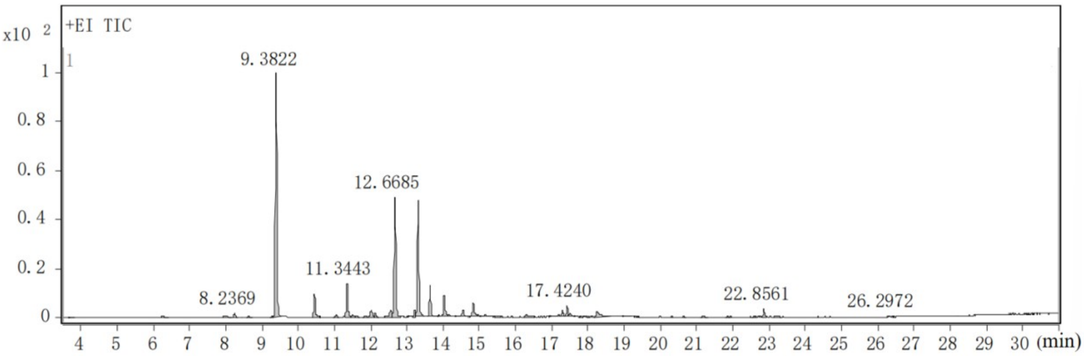

AA4 GC chromatogram

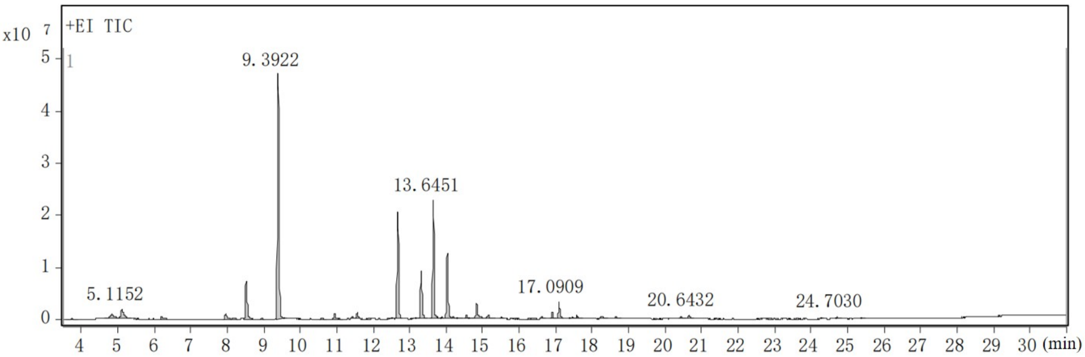

AL1 GC chromatogram
